# Prevalence of delivery mode in an Italian nationwide cohort with celiac disease: a SIGENP multicenter retrospective study (the CD-deliver-IT)

**DOI:** 10.1186/s13052-024-01710-0

**Published:** 2024-07-27

**Authors:** Donatella Iorfida, Francesco Valitutti, Annarita Vestri, Grazia D’Adamo, Tiziana Passaro, Marco Crocco, Federica Malerba, Alice Monzani, Ivana Rabbone, Licia Pensabene, Laura Giancotti, Francesco Graziano, Michele Citrano, Francesca Ferretti, Chiara Maria Trovato, Caterina Pacenza, Mario Iasevoli, Claudia Banzato, Riccardo Lubrano, Monica Montuori, the “Food-induced disease” study group of SIGENP, Luigi Principessa, Elisa D’Angelo, Basilio Malamisura, Angela Calvi, Noemi Zampatti, Ilaria Montafia, Antonella Diamanti, Pasquale Pisano

**Affiliations:** 1 Department of Maternal and Child Health, Pediatrics and Neonatology Unit, Santa Maria Goretti Hospital, Sapienza - University of Rome, Latina, Italy; 2 Department of Surgical and Biomedical Sciences, Pediatric Clinic, University of Perugia, Perugia, Italy; 3 Department of Public Health and Infectious Disease, Sapienza - University of Rome, Rome, Italy; 4 Pediatric Unit, AOU Salerno, P.O. Cava de’ Tirreni, Salerno, Italy; 5 Pediatric Gastroenterology and Endoscopy Unit, IRCCS Istituto Giannina Gaslini, Genoa, 16147 Italy; 6 Department of Neuroscience, Rehabilitation, Ophthalmology, Genetics, Maternal and Child Health, University of Genoa, Genoa, Italy; 7 Department of Health Sciences, Division of Paediatrics, University of Piemonte Orientale, Novara, Italy; 8 Department of Surgical and Medical Sciences, Pediatric Unit, Magna Graecia University, Catanzaro, Italy; 9 Pediatric Unit, Villa Sofia - Cervello Hospital, Palermo, Italy; 10 Hepatology Gastroenterology and Nutrition Unit, Bambino Gesù Children Hospital, Rome, Italy; 11 Pediatric Unit, “S. Giovanni di Dio” Hospital, Crotone, Italy; 12 Department of Medicine, Surgery and Dentistry “Scuola Medica Salernitana”, Pediatrics Section, University of Salerno, Baronissi, Italy; 13 Pediatric Division, University of Verona, Verona, Italy; 14 Maternal and Child Health Department, Pediatric Gastroenterology and Liver Unit, Sapienza - University of Rome, Rome, Italy

**Keywords:** Celiac disease, Mode of delivery, Cesarean section, Gut microbiota

## Abstract

**Background:**

Studies have indicated an association between cesarean section (CS), especially elective CS, and an increased risk of celiac disease (CD), but the conclusions of other studies are contradictory. The primary aim of this study (CD-deliver-IT) was to evaluate the rate of CS in a large population of CD patients throughout Italy.

**Methods:**

This national multicenter retrospective study was conducted between December 2020 and November 2021. The coordinating center was the Pediatric Gastroenterology and Liver Unit of Policlinico Umberto I, Sapienza, University of Rome, Lazio, Italy. Eleven other referral centers for CD have participated to the study. Each center has collected data on mode of delivery and perinatal period of all CD patients referring to the center in the last 40 years.

**Results:**

Out of 3,259 CD patients recruited in different Italian regions, data on the mode of delivery were obtained from 3,234. One thousand nine hundred forty-one (1,941) patients (60%) were born vaginally and 1,293 (40%) by CS (8.3% emergency CS, 30.1% planned CS, 1.5% undefined CS). A statistically significant difference was found comparing median age at time of CD diagnosis of patients who were born by emergency CS (4 years, CI 95% 3.40–4.59), planned CS (7 years, CI 95% 6.02–7.97) and vaginal delivery (6 years, CI 95% 5.62–6.37) (log rank *p* < 0.0001).

**Conclusions:**

This is the first Italian multicenter study aiming at evaluating the rate of CS in a large population of CD patients through Italy. The CS rate found in our CD patients is higher than rates reported in the general population over the last 40 years and emergency CS seems to be associated with an earlier onset of CD compared to vaginal delivery or elective CS in our large nationwide retrospective cohort. This suggests a potential role of the mode of delivery on the risk of developing CD and on its age of onset, but it is more likely that it works in concert with other perinatal factors. Further prospective studies on other perinatal factors potentially influencing gut microbiota are awaited in order to address heavy conflicting evidence reaming in this research field.

## Background

Celiac disease (CD) is an immune-mediated systemic disorder elicited by gluten and related prolamines in genetically susceptible individuals and characterized by the presence of a variable combination of gluten-dependent clinical manifestations, CD-specific antibodies, human leucocyte antigens (HLA)-DQ2 or HLA-DQ8 haplotypes, and enteropathy [1].

CD provides a unique model for autoimmune research, because the following key elements are known: the specific genes involved in its pathogenesis and the environmental trigger [2]. Although gluten consumption and certain HLA antigen genotypes are key factors for CD development, not all individuals with a predisposing genetic background develop loss of tolerance towards gluten [3, 4]: specifically, about 30% of the general population carry these genes [5], but only about 1% of the population develop CD [6]. This suggests that the risk is likely to be modified by other potential pathophysiological factors [7, 8], which are still to be clarified [9].

Several studies showed intestinal dysbiosis (altered gut microbiota composition or function) in patients with CD, either untreated or treated with a gluten-free diet [10–17]. Gut microbiota affects gut permeability and gut inflammatory activity (both directly and via the release of metabolites), which are suspected to play a role in increasing the risk of autoimmune disorders [18].

Mode of delivery is crucial for the acquisition of the microbiota after birth [19]. It has been hypothesized that infants delivered by cesarean section (CS) acquire different bacterial communities compared to infants born vaginally [19], with potential influence on the short and long-term immune responses to environmental factors, thereby predisposing to autoimmunity [18]***.*** Although different studies have demonstrated the difference in early microbiome development between delivery modes, the underlying pathogenetic mechanisms have not yet been identified.

Studies have indicated an association between CS, especially elective CS [20], and an increased risk of CD [21–24], but the conclusions of other studies on this topic are contradictory [25–29].

Given this background, the primary aim of this study (CD-deliver-IT) was to evaluate the rate of CS in a large population of CD patients throughout Italy.

## Methods

This national multicenter retrospective study was conducted between December 2020 and November 2021. The coordinating center was the Pediatric Gastroenterology and Liver Unit of Policlinico Umberto I, Sapienza, University of Rome, Lazio, Italy. Eleven other referral centers for CD have participated to the study (3 other centers located in Lazio, 2 respectively in Campania and in Calabria, 1 in each of these regions: Veneto, Liguria, Piedmont, Sicily).

Each center has collected data on mode of delivery and perinatal period of all CD patients referring to the center in the last 40 years.

These data were collected during planned follow up for CD or, alternatively, medical records were consulted to retrieve previously provided data. Collection of data through consultation of the Certificate of Delivery Assistance (CeDAP), which is the national source for vital birth information, was not possible. Required data were gender, date of birth, year of CD diagnosis, mode of delivery (elective CS or emergency CS or vaginal delivery), gestational age, birth weight, nationality and birthplace. Gestational age was defined as follows: preterm for babies delivered before 37 weeks of gestation, term birth for 37 0/7 weeks through 41 6/7 weeks, post-term if pregnancy has reached or extended beyond 42 0/7 weeks of gestation.

Unavailability of data relating the mode of delivery or unconfirmed CD diagnosis were considered exclusion criteria.

Continuous data were summarized by means (and standard deviation) and median (interquartile range). Categorical data were expressed as counts and percentages. The 95% confidence intervals of the rates was calculated (Wilson method). To compare continuous variables we performed t-test, while to compare categorical variables we used chi square test. We performed a Kaplan Maier method to evaluate the onset time of CD. The level of statistical significance was set at 0.05. All statistical analyzes were performed with STATA v.16.

To determine the sample size, it was assumed that the rate of CS is not very different from the rate at a national level; a sample of 1,372 subjects produced a 95% confidence interval, with a width equal to 5% when the hypothesized rate was equal to 32%.

Approximately 3000 patients have been screened in the study in order to identify the 1,372 subjects with the inclusion criteria, as per the planned size, for which all data have been collected according to CRF. Missing data imputation methods have not been used.

## Results

**Fig. 1:**
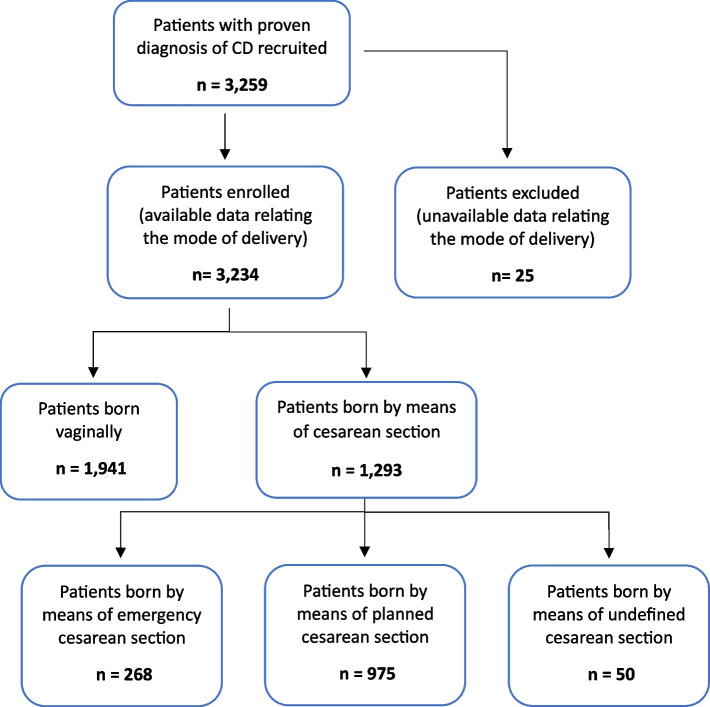
Study population. CD = celiac disease

Out of 3,259 patients with proven diagnosis of CD recruited in twelve Gastroenterology Services of different Italian regions, data on the mode of delivery were obtained from 3,234 patients (Table 1). Twenty-five subjects were excluded due to unavailable data relating the mode of delivery (Fig. 1).

**Table 1:** Italian regions of enrollment of patients included in the study

Region of enrollment	Number of patients enrolled
Campania	854 (26.4%)
Lazio	826 (25.5%)
Liguria	500 (15.5%)
Calabria	414 (12.8%)
Piedmont	322 (10%)
Sicily	262 (8.1%)
Veneto	56 (1.7%)

Out of 3,234 patients (M 1,164, 2 missing) age and age at time of CD diagnosis were known for 3,213 (99.4%) and 3,192 (98.7%) patients, respectively. Median age of patients included in the study was 14.0 years [IQR 9.0–18.0] and median age at time of CD diagnosis was 6.0 years [IQR 3.0–12.0].

**Fig. 2:**
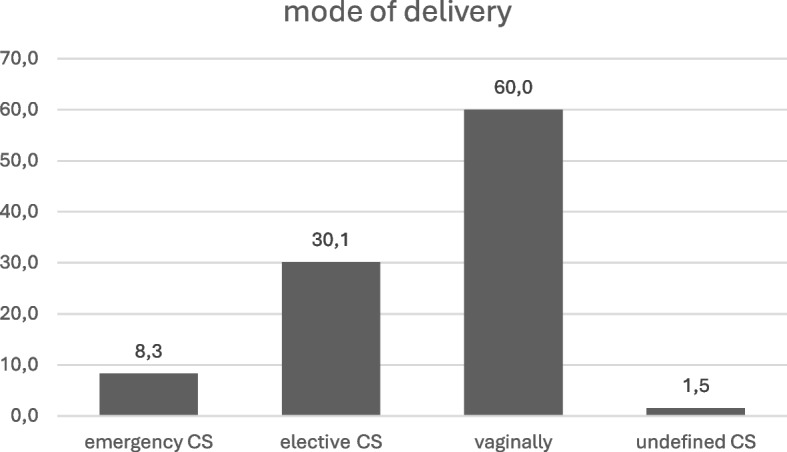
Mode of delivery of patients enrolled. CS = cesarean section

As regards the mode of delivery, 1,941 (60%) were born vaginally and 1,293 (40%) by CS (8.3% emergency CS, 30.1% planned CS, 1.5% undefined CS) (Figs. 1 and 2).

CS rate was also investigated by analyzing birth cohorts, and specifically: 1979–1989 (CS rate: 31%, CI95% 19.5–45), 1990–1999 (CS rate: 42.1%, CI95% 36.5–47.8), 2000–2009 (CS rate: 40.2%, CI95% 37.8–42.6) and 2010–2021 (CS rate: 39.5%, CI95% 36.7–42.3).

1745 newborn (54%) were born at term, 724 newborn (22.4%) were preterm, 263 newborn (8.1%) post-term; gestational age was unknown in 502 newborn (15.5%).

Nationality has been registered as Italian and foreign in 97.8% and 1.9% of patients respectively, whereas it was unknown in 0.3% of patients. Place of birth was recorded for 3,194 patients (98.8%) and it was a foreign state for 26 patients (0.8%); this data has not been retrieved for 40 (1.2%). Birthplace and region of enrollment were the same for 92% of patients.

**Fig. 3:**
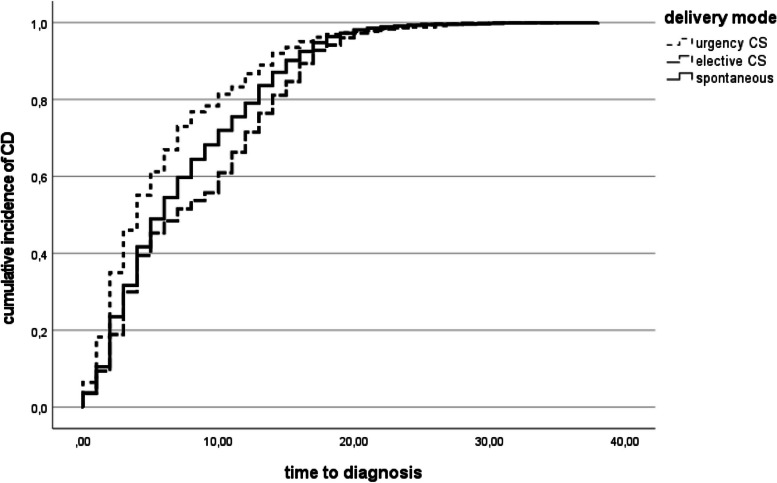
Median age at time of CD diagnosis when considering the type of CS. CS = cesarean section

With regards to median age at time of CD diagnosis, it was 6 years for patients delivered naturally (CI 95% 5.62–6.37) and 6 years for those born by CS (CI 95% 5.42–6.57). When considering the type of CS, a statistically significant difference was found comparing median age at time of CD diagnosis of patients who were born by emergency CS (4 years, CI 95% 3.40–4.59), planned CS (7 years, CI 95% 6.02–7.97) and vaginal delivery (6 years, CI 95% 5.62–6.37) (log rank *p* < 0.001) (Fig. 3).

Among patients who were born preterm, a statistically significant difference was found comparing median age at time of CD diagnosis of patients who were born by emergency CS (4 years, CI 95% 3.22–4.78) and those born by planned CS (6 years, CI 95% 4.76–7.23) (log rank *p* = 0.019).

When considering patients whose gestational age was at term, statistically significant differences with regard to median age at time of diagnosis were observed between patients born by emergency CS (3 years, CI 95% 2.05–3.94) and those born by planned CS (4 years, CI 95% 3.54–4.45) (log rank *p* = 0.001) and also when comparing patients born by emergency CS with those born by vaginal delivery (5 years, CI 95% 4.70–5.29) (log rank p = 0.001).

In post-term patients, median age at time of diagnosis was 4 years (CI 95% 2.41–5.58) in patients born by emergency CS and 12 years (CI 95% 9.11–14.88) in patients born by planned CS (log rank *p* < 0.001).

Considering data according to gender, statistically significant differences were found comparing median age at time of CD diagnosis both in males and females. Specifically, it was 4 years (CI 95% 2.82- 5.17) in males born by emergency CS and 7 years (CI 95% 5.33–8.66) in males born by planned CS (log rank *p* = 0.012). As regards females, a statistically significant difference was observed when comparing patients born by emergency CS (4 years, CI 95% 3.26–4.73) with those born by planned CS (7 years, CI 95% 5.56–8.43) (log rank *p* < 0.001) and with those born vaginally (6 years, CI 95% 5.51–6.48) (log rank *p* < 0.001).

## Discussion

Our study depicts the mode of delivery of a large dataset of CD patients followed-up at referral centers. The CS rate in our CD patients was found to be 40% (95% CI 38.2–41.6), a higher rate compared to the CS rates recorded in the last 40 years in Italy in the general population. In fact, although the frequency of CS has increased significantly in Italy in recent decades (11.2% in 1980, 27.9% in 1996, 33.2% in 2000, 37.5% in 2010, 31.2% in 2021) (CeDAP registry), with a slightly decreasing trend in last years, the CS rate observed among our CD patients is higher than the CS rate in the respective years even when examining specific birth cohorts. Whether the higher rate of CS observed in our CD population could suggest a potential pathogenetic role of CS in CD it is not possible to evaluate given our study design.

We found that emergency CS seems to be associated with an earlier onset of CD compared to vaginal delivery or elective CS in 3,234 patients with diagnosed CD. Specifically, an earlier diagnosis of CD was found in children born by emergency CS compared to planned CS in the analysis of all patients enrolled and also in all subgroup analysis: this result was found in preterm, at-term and post-term patients and in both males and females. An earlier CD diagnosis in children born by emergency CS compared to children born vaginally was found analyzing all patients enrolled and in two subgroup analysis (at-term born babies and females).

Although our results may seem controversial, considering that infants delivered by emergency CS are exposed to the maternal vaginal microbiota during birth as well as those born naturally, the dogma that difference in early microbiome development between delivery modes is due to lack of vaginal exposure was recently challenged in a study by Mitchell et al. [30]. The similarity between infants delivered by CS before or after labor in that study is consistent with findings in the Baby Biome Study [31]: these authors have reported disrupted maternal transmission of Bacteroides strains and high level colonization by healthcare-associated opportunistic pathogens in babies born by CS and in those delivered vaginally with maternal antibiotic prophylaxis or not breastfed during the neonatal period. Indeed, it is recognized that the gut microbiota composition can be perturbed by other early life events, including feeding and antibiotic exposure [24, 31].

It is therefore understandable why the literature framework on this topic is wide and, often, contradictory. Some studies reported an increased risk of CD in individuals born by CS [20, 21, 32–34], whereas other studies did not [6, 22, 25, 26, 28, 29, 35–37].

Specifically, studies reporting an increased risk of CD in individuals born by CS have shown different evidence as regards the type of CS (planned or emergency CS) [20, 21, 32–34].

In the retrospective, multicenter, case–control study by Decker et al. [21] a significantly enhanced likelihood of being born by CS was found in children with CD compared with control subjects. A register-based national cohort study by Andersen et al. [32] found an increased risk of diabetes, arthritis, CD, and inflammatory bowel disease after CS compared with vaginal delivery: in a subgroup analysis, both acute and elective CS was associated with an increased risk of developing a chronic inflammatory disease. A recent study by Maleki et al. [34] concluded that babies born vaginally have a lower risk of developing CD than children born by CS.

On the other hand, Marild et al. [20] found a positive association between planned CS and later CD, but no increased risk of CD following emergency or any CS. Also analysis of data from a large United States-based mother- child cohort [33] revealed that being born by CS without labor may be associated with an increased risk for CD, while infants born by CS with labor did not.

The conclusions of other studies on this topic are different. According to the results of an Italian population-based birth cohort study published in 2014 perinatal factors, including CS, have little influence on the risk of childhood CD; otherwise, use of antibiotics and gastrointestinal infections in the first year of life may facilitate the early onset of CD by altering intestinal microflora and the gut mucosal barrier [25, 28]. One year later the Norwegian Mother and Child cohort study concluded that CD was not associated with mode of delivery [22]. In 2018 Sander et al. published the results of a large registry-based study, showing that mode of delivery was not associated with an increased risk of diagnosed CD [6] and the Teddy study of the same year went in a similar direction, finding that CS was not associated with increased risk for CD in the offspring [29]. The mode of delivery did not influence the risk of developing CD neither in a study by Lionetti et al. involving a cohort of children genetically predisposed to CD and prospectively followed from birth [35].

No associations between CS and CD were found in the study by Sevelsted et al. [26] analyzing children born by CS for risk of chronic immune diseases. On the other hand, a study published in 2021 on a cohort study of over 900,000 children showed that CS was not associated with the risk of hospitalization for a range of autoimmune disorders before 14 years of age, suggesting that this mode of delivery may not be related to the development of autoimmunity [36].

To summarize comprehensive data available, a recent systematic review and meta-analysis found that, compared with spontaneous birth, CS was not associated with an increased risk of CD and in subgroup analyses the association remained non-significant for both infants born after planned CS and emergency CS [37]. Authors underlines that all of studies in this meta-analysis did not report antibiotic exposure and thus they are unable to eliminate influences antibiotic on risk of CD. Moreover, they suggest that investigation of the association between CS and CD in children should take also breastfeeding into consideration. In addition to this, authors recognize that this meta-analysis is limited by the inclusion of exclusively observational studies, which are susceptible to confounding and that inadequate control for potential confounders may bias the results.

Among these studies with conflicting results, a potential explanation of an earlier diagnosis of CD in children born by emergency CS in our study could be found considering also other perinatal factors besides CS. Breastfeeding seems to moderate the effects of CS [38–40] and intrapartum antibiotics [41] on the early microbiota, producing a microbiota profile more similar to that of infants born vaginally or those not receiving antibiotics [42, 43]*.* Duration of breastfeeding is usually shorter in patients born by means of emergency CS compared to patients born by scheduled CS or spontaneous birth [44] and different studies have shown that prolonged breastfeeding is associated with a postponed diagnosis of CD [45–48]*.* Anyway, this perinatal factor was not investigated in our study.

Moreover, it might be reasonable to assume that emergency CS could imply an increased risk of infection, related to the indication for the procedure, for instance, the occurrence of an infection in the fetus or labor that has been prolonged, as well as the procedure itself [49]*.* The increased risk of infection entails a more frequent use of antibiotics in these babies and/or laboring mothers, and it is reported that exposure to early infections or antibiotics is associated to subsequent CD [50, 51]. Nevertheless, this hypothesis is not possible to be confirmed by our study design.

To our knowledge, this is the first Italian multicenter study aiming at evaluating the rate of CS in a large population of CD patients through Italy. The large population enrolled and the speed data collection represent strengths of this study. Moreover, only 0.8% of patients enrolled were born abroad, while the majority of CD patients included in this study were born in Italy (regions of Northern, Central and Southern of Italy), so data of this study could be applied to the whole Italian territory.

Nevertheless, we are aware of some limitations. The lack of information about other potential confounding factors, including perinatal and maternal factors, such as perinatal use of antibiotics, perinatal infections, duration of breastfeeding, family history for CD and social background is a limitation of our study. Moreover, data relating to the type of CS (planned or emergency CS) and to the gestational age were not available in 1.5% and 15.5% of patients respectively, although these percentages are low.

## Conclusions

In conclusion, the CS rate found in our CD patients is higher than rates reported in the general population over the last 40 years and emergency CS seems to be associated with an earlier onset of CD compared to vaginal delivery or elective CS in our large nationwide retrospective cohort. This suggests that mode of delivery could have a potential role on the risk of developing CD and on its age of onset, but it is more likely that it works in concert with other perinatal factors. Further prospective studies on other perinatal factors potentially influencing gut microbiota are awaited in order to clarify this intriguing topic and to address heavy conflicting evidence reaming in this research field.

## Data Availability

The datasets used and/or analysed during the current study are available from the corresponding author on reasonable request.

## Abbreviations

CS: Cesarean section
CD: Celiac disease
HLA: Human leucocyte antigens
CeDAP: Certificate of Delivery Assistance
